# Preventive Inoculation against Pleuro-pneumonia1Read before the June meeting of the Missouri Valley Veterinary Medical Association.

**Published:** 1896-08

**Authors:** E. Biart

**Affiliations:** Lansing, Kansas


### PREVENTIVE INOCULATION AGAINST PLEURO-PNEUMONIA.[1]

BY E. BIART, V.S.,
LANSING, KANSAS.

THE first really scientific way of preventing the spread of contagious diseases among cattle was discovered by Dr. Willems, of Hasselt, Belgium, as early as 1852, but was only brought into actual practice in 1881. It was established on a solid basis in his laboratory (the stables of a large distillery). The young doctor inoculated directly into the steer the contagious pleuro-pneumonia virus, injected into the muscle or the subcutaneous cellular tissues. The germ produced, in addition to the local tumor, phenomena of severe intoxication, from which the animal died in a very short time. The doctor then decided to inoculate the same virus at the lower extremity of the caudal appendix. Then, again, the symptoms soon made their appearance after the insertion of the virus, but were of a mild type, and the animal soon recovered. It was then proved that the animal had become insusceptible to the disease, for, although being reinoculated with the virus several times, and in all regions of the body, there was no more sign of reaction, and the animal did not show any of the symptoms of the disease.

Dr. Willems first isolated the contagious pleuro-pneumonic microbe, and with the virus inoculated dogs, cats, horses, and other animals, and even human beings, without in a single instance producing the least inconvenience or symptoms of the disease; but when inoculated to cattle they would invariably show unmistakable signs of the disease. The disease, it was hereby proven, was consequently confined to the bovine race, not being communicable to other animals, whether ruminants or solipeds, nor even to man. From this discovery it resulted that the opinion of scientists generally was: That the flesh of animals slaughtered in the first stage of the disease is not deleterious to the public health, and can with perfect safety be used as food.

The virus was now discovered, but it was so violent that one drop of it, injected under the skin, would invariably cause

[1] Read before the June meeting of the Missouri Valley Veterinary Medical Association.

death; and it appeared that such a severe poison could never become a beneficent vaccine.

The discoverer resumed his work, and when after years of laborious researches he finally found the best method of gathering the vaccine, the period of the disease where it is at the same time the most active and the most innocuous, and the place of the body where you can safely inoculate, he generously gave the government the benefit of his experiences, without asking even for a reward.

The Department of Agriculture now appointed a committee of noted veterinarians to investigate the preventive mode of treatment. They consequently repaired to the doctor's home, whose stables were claimed to be proof against the disease, he having inoculated all of the animals with the pleuro-pneumonia virus. The doctor being asked to have introduced in his stable and mixed with his cattle other cattle manifestly diseased, and this in all stages of the malady, readily consented, and, although they were kept in constant and close proximity with one another for a great length of time, none of the inoculated cattle contracted the disease, nor did they show the least inconvenience.

Switzerland and Italy almost immediately accepted the system, which afterward proved effective and of great value. The practice is now generally adopted, and in those countries, wherever employed, contagious pleuro-pneumonia is almost a thing of the past. In Belgium, where the method originated, not a case has been discovered in the last two years.

*Modus operandi.* The serum which floats on the expressed fluids of diseased lungs, in the first or second stages of the disease, and this must be as fresh as possible, is the right virus for inoculation. The inoculation is done on the lateral faces of the extremity of the tail, by two incisions, about two inches apart. The skin is then raised and a small amount of the serum injected, the same as vaccination in the human; a few drops is sufficient. The animal can be turned out without further care; but when local inflammation becomes too intense, from fourteen to twenty ounces of salts must be given. Generally after ten days of inoculation, seldom before, and at the point of inoculation, will be seen an inflammatory engorgement, hard, hot, and very sensitive, very circumscribed, but in exceptional cases extending to a vast area of tissue, and this generally becomes gangrenous. In ordinary cases this engorgement gives rise to pustules, throwing out serum and covered with scabs, which dry up, but often fall off and leave an ulcerous wound, which finally heals completely without care. In order to have a successful inoculation these pustules should appear, or at least the inflammatory engorgement. Animals in which no reaction has taken place should be inoculated in from three to four weeks later. Inoculated animals must be closely watched from the fifth or sixth day after the operation, and as soon as tumefaction is seen at the seat of inoculation a long, deep incision should be made, and this should bleed freely and suppuration encouraged. If it is noticed that the end of the tail becomes gangrenous, it should be amputated till in the sound portion, as this is the only way to stop the invading process of the disease. By carefully carrying out the above instructions failures are almost impossible.

On to Buffalo!

